# Measuring Neural Entrainment to Beat and Meter in Infants: Effects of Music Background

**DOI:** 10.3389/fnins.2016.00229

**Published:** 2016-05-24

**Authors:** Laura K. Cirelli, Christina Spinelli, Sylvie Nozaradan, Laurel J. Trainor

**Affiliations:** ^1^Department of Psychology, Neuroscience and Behaviour, McMaster UniversityHamilton, ON, Canada; ^2^MARCS Institute, Western Sydney UniversityMilperra, NSW, Australia; ^3^Institute of Neuroscience, Université Catholique de LouvainLouvain-la-Neuve, Belgium; ^4^BRAMS, Université de MontréalOutremont, QC, Canada; ^5^McMaster Institute for Music and the Mind, McMaster UniversityHamilton, ON, Canada; ^6^Rotman Research Institute, Baycrest HospitalToronto, ON, Canada

**Keywords:** neural entrainment, rhythm, meter, electroencephalography, infancy, steady-state evoked potentials, music, frequency-tagging

## Abstract

Caregivers often engage in musical interactions with their infants. For example, parents across cultures sing lullabies and playsongs to their infants from birth. Behavioral studies indicate that infants not only extract beat information, but also group these beats into metrical hierarchies by as early as 6 months of age. However, it is not known how this is accomplished in the infant brain. An EEG frequency-tagging approach has been used successfully with adults to measure neural entrainment to auditory rhythms. The current study is the first to use this technique with infants in order to investigate how infants' brains encode rhythms. Furthermore, we examine how infant and parent music background is associated with individual differences in rhythm encoding. In Experiment 1, EEG was recorded while 7-month-old infants listened to an ambiguous rhythmic pattern that could be perceived to be in two different meters. In Experiment 2, EEG was recorded while 15-month-old infants listened to a rhythmic pattern with an unambiguous meter. In both age groups, information about music background (parent music training, infant music classes, hours of music listening) was collected. Both age groups showed clear EEG responses frequency-locked to the rhythms, at frequencies corresponding to both beat and meter. For the younger infants (Experiment 1), the amplitudes at duple meter frequencies were selectively enhanced for infants enrolled in music classes compared to those who had not engaged in such classes. For the older infants (Experiment 2), amplitudes at beat and meter frequencies were larger for infants with musically-trained compared to musically-untrained parents. These results suggest that the frequency-tagging method is sensitive to individual differences in beat and meter processing in infancy and could be used to track developmental changes.

## Introduction

Mothers across cultures interact with their infants in musical ways, frequently singing them lullabies and playsongs (Trehub and Schellenberg, 1995; de l'Etoile, 2006; Trehub and Gudmundsdottir, 2015). In turn, infants respond positively to this input (Trainor, 1996). Furthermore, caregivers rock infants to the rhythms of music, and such synchronous interpersonal movement appears to increase infant social affiliative behaviors (Cirelli et al., 2014a,b; 2016; Tunçgenç et al., 2015). Yet little is known about how infants' brains encode musical rhythms. The present paper reports the results of two experiments using an original electroencephalographic (EEG) frequency-tagging approach to investigate the neural encoding of rhythms in 7- and 15-month-old infants. The results suggest that the frequency-tagging approach can be successfully used with infants, and also revealed individual differences in musical rhythm processing related to differences in infant and parent music training.

Humans are very good at organizing timing structures in music (for a review, see Repp and Su, 2013). From the rhythm (i.e., pattern of tone onsets and offsets), people can easily extract the underlying pulse, or *beat*. These beats are then perceptually organized into hierarchical groups to create an internal *metrical* structure representation through which the musical input is interpreted as alternating patterns of strong and weak beats. Some examples of common Western music meters include grouping isochronous beats into a duple metrical structure (groups of 2), a triple metrical structure (groups of 3), or a quadruple metrical structure (groups of 4). While non-musicians easily perceive meter (especially when low-level components of the rhythm make meter salient), musicians often display advantages on tasks involving meter perception and production (for example, Drake et al., 2000; Brochard et al., 2003). Perception of beat and meter are not only driven by auditory cues in the stimulus, but also shaped by top-down processes such as attention, expectation and previous experience (see for example Large and Jones, 1999; Brochard et al., 2003; Phillips-Silver and Trainor, 2007, 2008; Nozaradan et al., 2011; Schaefer et al., 2011; Manning and and Schutz, 2013; Chemin et al., 2014; Butler and Trainor, 2015; Celma-Miralles et al., 2016). For example, when listening to unaccented isochronous tones, both musician and non-musician adults showed larger event-related potentials (ERPs) to every second tone, suggesting that a duple metrical structure was automatically applied (Brochard et al., 2003). Thus, meter perception can be automatic, especially when low-level information in the rhythmic stimulus increases the salience of strong over weak metrical beats, but it can also be shaped by attention and music training.

While many questions remain about the developmental time-course of beat and meter perception, there is evidence that young infants are sensitive to this timing information. Newborns can discriminate between spoken languages that fall into different rhythmic categories (for example, English compared to Japanese) (Nazzi et al., 1998). By as early as 2 months of age, infants can detect tempo changes (Baruch and Drake, 1997) and discriminate between different musical rhythm patterns (Chang and Trehub, 1977; Demany et al., 1977). In terms of meter processing, Winkler et al. (2009) argue that such processing occurs even in newborn infants. Using electroencephalography (EEG) and a mismatch-negativity paradigm, they showed that newborns were better able to detect the omission of a metrically important than metrically unimportant beats in a rhythm pattern. Behaviorally, by as early as 7 months of age infants can categorize melodies and rhythms based on metrical structure (Hannon and Johnson, 2005), and use movement to guide meter perception (Phillips-Silver and Trainor, 2005).

Infants are also learning about metrical structure through exposure to music during the first year after birth. Hannon and Trehub (2005a,b) revealed this effect of exposure by taking advantage of the fact that musical systems in different cultures use predominantly different meter styles. For instance, Western meters tend to be simple, with metrical groupings of beats by 2 or 3, whereas music from many other places in the world contains complex meters with metrical groupings of 5 or 7 beats (for example, Bulgarian music). Hence, Western adults are much better at detecting violations in patterns with simple meters compared to patterns with complex meters, whereas adults exposed to musical systems containing complex meters (such as Bulgarian music) are equally good at detecting violations in patterns with both simple and complex meters (Hannon and Trehub, 2005a). Interestingly, at 6 months of age, Western infants are apt at detecting metrical violations in both culturally familiar and unfamiliar rhythmic patterns. However, by the time these babies are 12-months-old, they perform like adults, and are only able to detect violations in patterns with simple meters (Hannon and Trehub, 2005b). This perceptual narrowing indicates that musical exposure during the first year after birth shapes how musical timing structures are processed and perceived by infants.

The influence of controlled musical exposure during infancy on music processing has not been extensively studied, but one series of experiments did find evidence for such effects (Gerry et al., 2012; Trainor et al., 2012). In this investigation, 6-month-old infants and their parents were randomly assigned to attend 6-months of one of two types of caregiver/infant classes: (1) active music classes or (2) control classes focusing on play while music was presented passively. After (but not before) the training period, infants in the active music classes displayed larger and earlier brain responses to musical sounds as measured using EEG. Interesting correlations between preferences for expressive over mechanical music performances and socio-economic status (SES) were also found, independent of class assignment. Infants from families with a higher compared to lower SES were more likely to prefer expressive music to synthesized non-expressive music (Trainor et al., 2012). While this correlation is difficult to interpret, it is possible that parents from a higher SES have the means to receive music training themselves and expose their infants to a wider variety of musical stimuli. While these results have important implications on how musical exposure in infancy shapes music perception, they do not address how experience might affect the encoding of beat and meter in infancy.

One promising method for exploring infant rhythm processing is the EEG frequency-tagging approach (see Nozaradan, 2014 for a review). This original method was initially used to investigate the neural mechanisms underlying rhythm processing in adults. Neural entrainment to the incoming rhythm is measured in the form of peaks emerging from the EEG spectrum at frequencies corresponding to the rhythm envelope (Nozaradan et al., 2012b). In an initial study, participants were asked to listen to an isochronous auditory stimulus with a 2.4 Hz beat frequency and to imagine either that beats were metrically grouped in twos (a duple meter frequency at 1.2 Hz) or threes (a triple meter frequency at 0.8 Hz) (Nozaradan et al., 2011). The sound stimulus itself did not contain any energy at either of these metric frequencies. Interestingly, compared to when they were not asked to imagine a metrical structure, participants displayed a peak of brain activity (i.e., steady-state evoked potentials, or SS-EPs) specifically located at the imagined metrical frequencies. Importantly, this result suggests that the SS-EPs elicited in response to the sound do not merely constitute a faithful encoding of the stimulus rhythm. Rather, the brain transforms the rhythmic input by amplifying frequencies that coincide with perceived beat and meter frequencies. This finding was corroborated by subsequent frequency-tagging studies showing that SS-EPs elicited at frequencies corresponding to the perceived beat and meter were influenced not only by bottom-up stimulus properties, but also by top-down processes such as movement or predictive timing (Chemin et al., 2014; Nozaradan et al., 2012b, 2015, 2016).

The purpose of the present investigation was to use the frequency-tagging approach with infants to test whether music training shapes the neural encoding of rhythms early in infancy. EEG was recorded while infants listened to rhythmic patterns. There were two main goals of the research. First and foremost, we aimed to investigate the neural entrainment to rhythmic patterns in infants by measuring SS-EPs at beat- and meter-related frequencies in the EEG spectrum. Second, we investigated how individual differences in music background correlated with individual differences in these SS-EP measurements. We expected to find enhanced neural entrainment at beat and/or meter frequencies in the infants with stronger music backgrounds. Information was collected about parents' music training, enrolment in caregiver/infant music classes, and weekly hours of music listening. Experiment 1 presents results from a large sample of 7-month-old infants. These babies listened to an ambiguous rhythmic stimulus (that could be interpreted as in either duple or triple meter) used previously in behavioral studies with infants and adults (Phillips-Silver and Trainor, 2005, 2007), as well as EEG studies with adults (Chemin et al., 2014). By this age, infants are not yet encultured to their musical environment, but do perceive beat and meter (Hannon and Trehub, 2005a). Experiment 2 presents results from 15-month-old infants, some whom had been recently randomly assigned to attend caregiver-infant music classes. These older infants listened to an unambiguous rhythmic stimulus with a typical Western quadruple meter that had been previously used in behavioral and EEG studies with adults (Nozaradan et al., 2012a, 2016). By this age, infants should be encultured to their musical environment, and should show more adult-like responses. Having two age groups and two different rhythm patterns provides a test of the generalizability of the frequency tagging method.

## Experiment 1

### Materials and methods

#### Participants

Sixty 7-month-old (28 males; M age = 7.56 mo, SD = 0.29 mo) normal hearing infants participated in this experiment. An additional 14 infants participated, but were too fussy to complete the procedure. These infants were recruited from the Developmental Studies Database at McMaster University. The McMaster Research Ethics Board approved all procedures and informed consent was obtained from parents.

#### Stimulus

**Figure 1:**
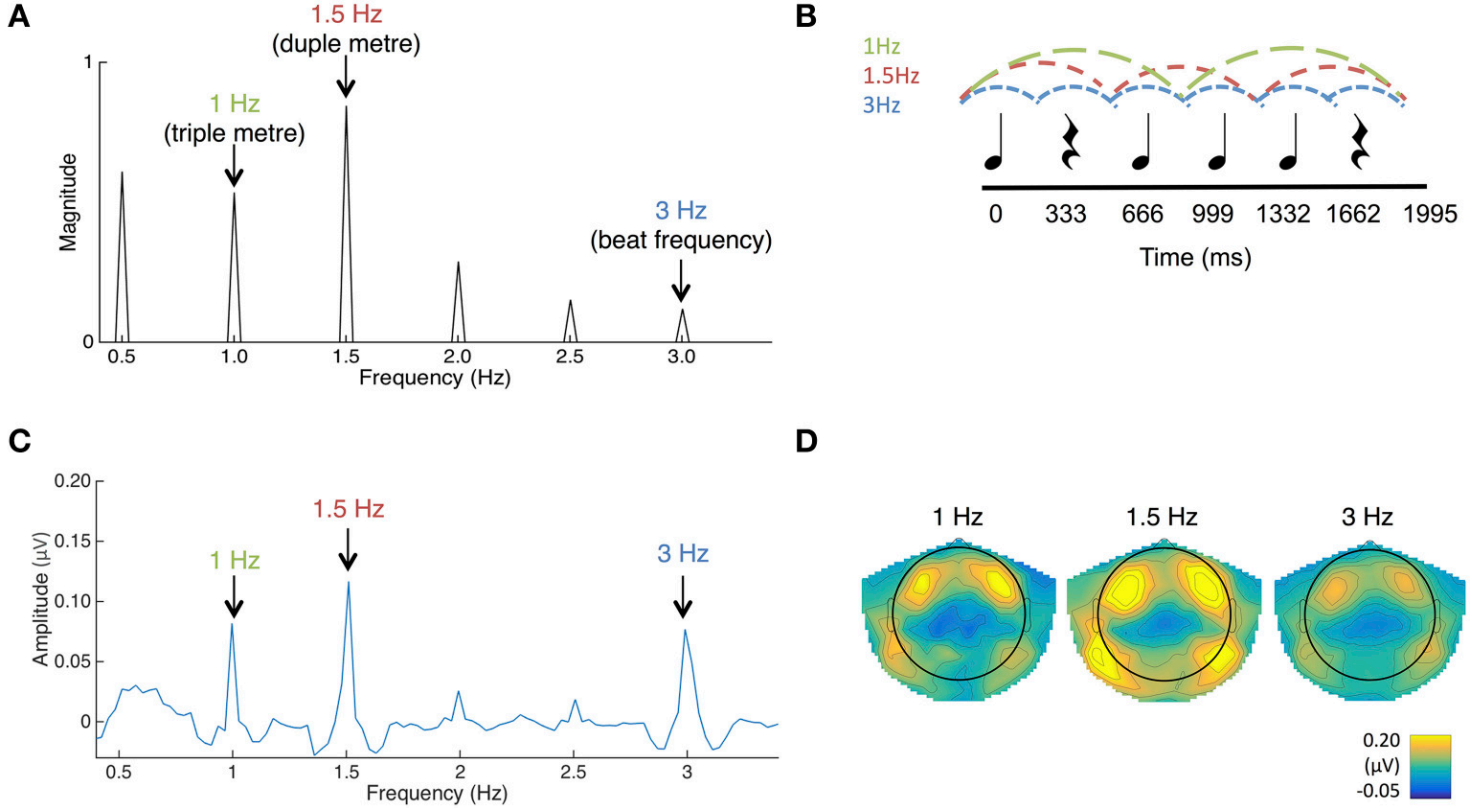
**(A)** The frequency spectrum of the stimulus sound envelope. **(B)** The rhythmic pattern, which consisted of 333 ms long tones and rests. Each tone consisted of 990 Hz pure tones with 10 ms rise and fall times. **(C)** Average SS-EPs with noise subtracted amplitudes averaged across all channels. Peaks can be visualized at the triple meter frequency (1 Hz), duple meter frequency (1.5 Hz), and beat frequency (3 Hz). **(D)** Average topographical map of the SS-EPs elicited at the triple meter frequency (1 Hz), duple meter frequency (1.5 Hz), and beat frequency (3 Hz), averaged across all participants.

The stimulus consisted of a six-beat rhythm pattern, lasting 2 s, based on the stimulus used by Phillips-Silver and Trainor (2005) (Figure 1B). The rhythm pattern consisted of the following: tone-silence-tone-tone-tone-silence. Each beat had an inter-onset-interval of 333 ms (180 beats per minute), which translated to a beat frequency of 3 Hz. The tones were 990 Hz pure tones lasting 333 ms with 10 ms rise and fall times synthesized using the program Audacity 2.0.5 (www.audacity.sourceforge.net). One 34-s long trial consisted of 17 repetitions of this stimulus. These trials were repeated 32 times, with no pauses between trials, so that the entire procedure lasted just over 18 min with a break at the halfway point. The stimulus was presented at a comfortable intensity level [~60 dB SPL at the location of the infants' head over a noise floor of < 30 dB(A)] using Eprime software through an AudioVideo Methods speaker (P73) located approximately 1 m in front of the infant.

To determine frequencies of interest for the SS-EP analysis, the temporal envelope of the rhythm pattern was extracted using a Hilbert function implemented in MATLAB, yielding a time-varying estimate of the instantaneous amplitude of the sound envelope. The obtained envelope was then transformed in the frequency domain using a discrete Fourier transform, yielding a frequency spectrum of acoustic energy (Figure 1A).

#### Procedure

After the nature of the study was described, the infant's parent(s) gave written consent to participate and also filled out a questionnaire about their child's and their own hearing and musical history.

The parent sat on a chair ~1 m in front of the speaker, and held their infant on their lap. Infants' EEG signals were recorded while they passively listened to the stimulus for 18 min, with one break at the halfway point. During the procedure, an experimenter stayed in the room and silently entertained the infant with puppets, bubbles and toys to keep them still and content. A silent video played on a monitor below the speaker. Parents were asked to not speak during the recording session and to minimize their movements.

#### Data acquisition and analysis

EEG signals were collected using a 124-channel HydroCel GSN net with an Electrical Geodesic NetAmps 200 amplifier passing a digitized signal to Electrical Geodesics NETSTATION software (v.4.3.1). Signals were recorded online with at a sampling rate of 1000 Hz and with a Cz reference. Electrode impedance during recording was maintained below 50 kΩ.

The data were filtered offline using EEProbe Software with high-pass and low-pass filters set at 0.5 and 20 Hz respectively. The data were resampled at 200 Hz in order to be processed using the Artifact Blocking algorithm in MATLAB (Mourad et al., 2007). This algorithm is especially useful for improving signal to noise ratios in continuous infant data (Fujioka et al., 2011). Using EEProbe Software, recordings were then digitally re-referenced to a common average. The 32 trials were averaged from 1000 to 34,000 ms, with baseline defined between 900 and 1000 ms. The first second of each epoch was removed (i) to discard the transient auditory evoked potentials related to stimulus onset and (ii) because SS-EPs require several cycles of stimulation to be entrained (Regan, 1989; Nozaradan et al., 2011, 2012b).

A Fourier transform was applied to the averaged EEG waveforms at each electrode using Letswave5 (Mouraux and Iannetti, 2008). This yielded a frequency spectrum where the signal amplitude (μV) ranged from 0 to 500 with a frequency resolution of 0.031 Hz. To obtain valid estimates of SS-EPs, the contribution of unrelated residual background noise was removed. This was accomplished by subtracting the averaged amplitude measured at neighboring frequency bins from each frequency bin (Mouraux et al., 2011; Nozaradan et al., 2012a). The two neighboring bins ranged from −0.15 to −0.09 Hz and +0.09 to +0.15 Hz relative to each frequency bin, thus corresponding to −3 to −5 and +3 to +5 bins around each frequency bin of the spectrum. Then, SS-EP magnitudes were averaged across all scalp electrodes for each participant, to allow SS-EP amplitudes to be compared across groups while avoiding electrode selection bias (Nozaradan et al., 2011, 2012b, 2016).

Event related potential (ERP) analyses were also performed on the filtered (0.5 Hz high pass, 20 Hz low pass), resampled, artifact corrected and re-referenced data. Epochs from −100 to 300 ms relative to the onset of the first tone in each 6-beat sequence were averaged (total trials = 544), with baseline defined as −100 to 0 ms. Waveforms from eight right frontal channels were averaged and waveforms from the corresponding eight left frontal channels were averaged to examine the response from auditory cortex. Because of the orientation of auditory cortex around the Sylvian Fissure, activity from auditory areas typically shows up at frontal electrode sites on the surface of the scalp (Trainor, 2012). We defined the time-point at which the largest magnitude peak occurred in the grand average at each electrode grouping. Area under the curve was then calculated for each individual infant for each hemisphere as the area ±50 ms around this time point.

### Results

To check for outliers, an average SS-EP amplitude score was calculated for each infant across the 5 peaks frequency-tagged from the sound stimulus (1, 1.5, 2, 2.5, and 3 Hz). The z-scores across these averages were calculated, and one infant was excluded from further analyses using a z-score cutoff of ±3. For ANOVAs using repeated measures, Greenhouse-Geisser corrections are reported where applicable.

#### SS-EP responses

SS-EPs averaged across all channels and scalp topographies are visualized in Figures 1C,D. The expected beat frequency is at 3 Hz (i.e., 333 ms long tones and rests in the rhythmic pattern). Based on previous work with this rhythm pattern (Phillips-Silver and Trainor, 2005, 2007; Chemin et al., 2014), this ambiguous stimulus pattern can be interpreted as in either duple or triple meter, although there is a bias in Western adults for the duple interpretation (Chemin et al., 2014). 1.5 Hz represents the related metrical frequency where beats are grouped in two (duple) and 1 Hz represents the metrical frequency where beats are grouped in three (triple).

To determine if the peaks in the frequency-transformed EEG occurred as expected above the noise floor, peaks of interest were first determined from the FFT of the sound stimulus (See Figure 1A). Amplitudes in the frequency-transformed EEG were calculated at frequencies where peaks were present in the sound stimulus (1, 1.5, 2, 2.5, and 3 Hz; 0.5 Hz was excluded due to our use of a 0.5 Hz high pass filter). These were also calculated at frequencies where no peaks were present in the sound stimulus (0.75, 1.25, 1.75, 2.25, and 2.75 Hz). These amplitudes were calculated by selecting the maximum amplitude within a 3-bin band centered on the frequency of interest.

Average noise floor amplitude was calculated as the average across 0.75, 1.25, 1.75, 2.25, and 2.75 Hz. Using paired-samples *t*-tests corrected for multiple comparisons using the Bonferroni correction, EEG amplitudes at each of the frequencies contained in the sound stimulus were significantly above this average noise floor (all *p*'s < 0.010). In addition, the average amplitude of beat and meter-related frequencies (1, 1.5, 3 Hz) was significantly greater than the average amplitude of beat- and meter-unrelated frequencies present in the sound (2 Hz, 2.5 Hz), *t*_(58)_ = 9.54, *p* < 0.001. The significant presence of peaks at both 1.0 and 1.5 Hz in the grand average likely reflects that some infants perceived the rhythm in duple meter and some in triple meter, or that individual infants may have switched back and forth in their interpretation, but we are not able to distinguish these possibilities. In general, these results suggest that the frequency tagging SS-EP method can result in significant signal to noise ratios when used with this age group.

#### Effects of music background

##### Effect of infant music classes

Thirteen infants in this sample were reported to have participated in infant music classes with their caregiver. Most (11 of the 13) reported attending the classes for 45–60 minutes hour per week. Classes were varied (e.g., Kindermusik, Music Together) and started at various ages (starting age ranged from 1- to 6-months-old).

**Figure 2:**
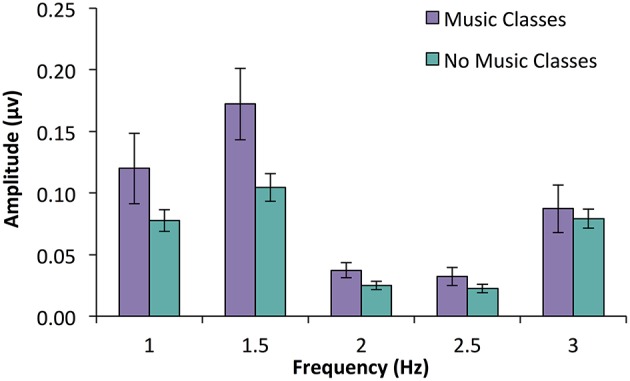
**SS-EP amplitudes (noise subtracted, averaged across all channels) across frequencies of interest for 7-month-old infants who have participated in parent-caregiver music classes (***n*** = 13, shown in purple) and infants who have not (***n*** = 46, shown in blue)**. Infants who have had music classes show larger amplitudes at the duple meter frequency (1.5 Hz) compared to those who do not, *p* = 0.012. Interestingly, there was no significant difference at beat frequency (3 Hz), *p* = 0.656.

An ANOVA with participation in music classes as a between subjects variable and frequency (five levels: beat, 3 Hz; duple meter, 1.5 Hz; triple meter, 1 Hz; unrelated, 2 and 2.5 Hz) as a within subjects variable was used to investigate SS-EP amplitudes. A main effect of infant music classes [*F*_(1, 57)_ = 5.692, *p* = 0.02] was qualified by a significant interaction between class participation and frequency, *F*_(2.83, 161.17)_ = 2.95, *p* = 0.037. We explored this interaction using *post-hoc t*-tests (using a Bonferroni correction and family-wise alpha of *p* = 0.10) to investigate how infants with music classes compared to those without at each frequency level. While infants with music classes did not have larger SS-EPs at the beat frequency (*p* = 0.656), triple meter frequency (*p* = 0.183), or either of the unrelated frequencies (*p* = 0.082 for 2 Hz; *p* = 0.216 for 2.5 Hz), infants with music classes did have larger amplitudes at duple meter frequency (1.5 Hz) than those without training, *t*_(57)_ = 2.58, *p* = 0.012 (See Figure 2).

**Figure 3:**
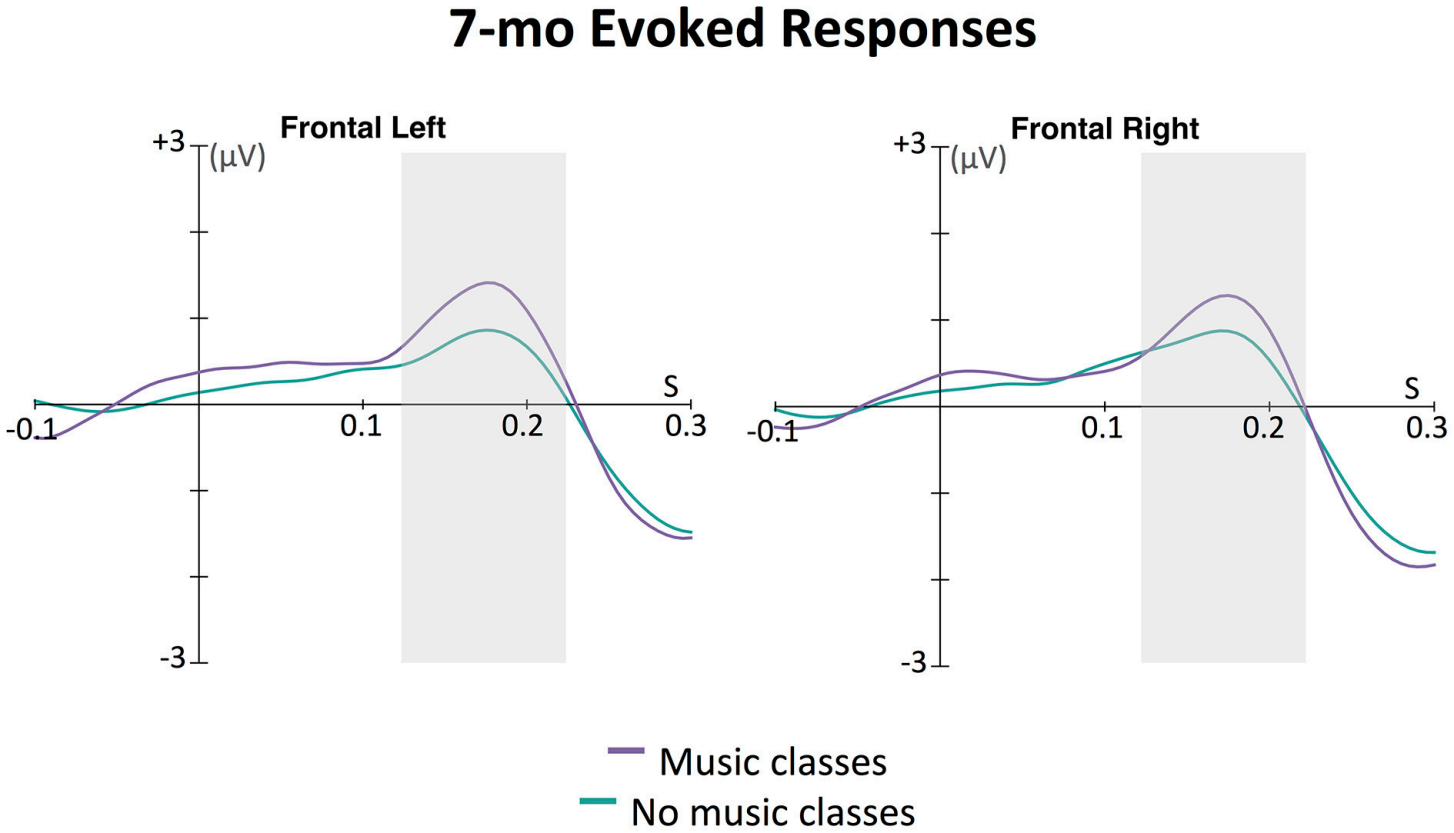
**Grand average evoked responses (filters: 0.5 Hz highpass, 20 Hz lowpass) by 7-month-old infants to first tone in the 6-beat sequence averaged across eight left frontal channels and eight right frontal channels**. The magnitude of the response for each group for each hemisphere was calculated as the area under the curve in a window ±50 ms around 175 ms (shown with the gray boxes); 175 ms was the time point of the largest amplitude peak in grand average of the slow positive evoked responses. In an ANOVA with infant music class enrollment as a between-subjects factor and hemisphere (left, right) as a within-subjects factor, only a main effect of music class enrollment was found. Infants who had attended music classes showed larger positive evoked responses to the sounds than infants who had not attended such classes, *p* = 0.048.

The effect of music classes was further explored in the ERPs to the first tone in each 6-beat sequence. At this age, infant ERP responses to tones are dominated by a slow positive wave at frontal electrodes, thought to be generated in auditory cortex (e.g., Leppänen et al., 1997; Trainor et al., 2003; He et al., 2007, 2009). As can be seen in Figure 3, this wave peaked around 175 ms after tone onset. The area under the curve method described above was used to assess the magnitude of this auditory response. An ANOVA was conducted with between-subjects factor infant music training and within-subjects factor hemisphere (left, right). While there was no main effect of hemisphere [*F*_(1, 57)_ = 0.01, *p* = 0.929] or interaction between hemisphere and infant music training [*F*_(1, 57)_ = 1.60, *p* = 0.211], there was a significant main effect of infant music training, *F*_(1, 57)_ = 4.09, *p* = 0.048. Infants who had been enrolled in music classes had larger evoked responses to these tones compared to those who had not been enrolled in such classes.

##### Effects of parent music training

Years of parent music training were calculated as a combination of mother- and father-reported levels. Infants were divided into two groups based on this information: infants with parents who had ≥5 years of combined music training (*n* = 24), and infants with parents who had < 5 years of music training (*n* = 35)

An ANOVA with parent music training groups as a between subjects variable and frequency (five levels: beat, 3 Hz; duple meter, 1.5 Hz; triple meter, 1 Hz; unrelated, 2 and 2.5 Hz) as a within subjects variable was used to investigate SS-EP amplitudes. There was no main effect of parent music classes [*F*_(1, 57)_ = 1.523, *p* = 0.222] and no interaction between music classes and frequency [*F*_(2.73, 155.56)_ = 0.41, *p* = 0.802]. There was also no correlation between reported years of parent music training and amplitudes at beat and meter frequencies (all *p*'s > 0.102).

##### Reported hours of infant music listening

Parents were asked to report how many hours a week their infants heard music (either passive or active, but while awake). These reported rates did not correlate with amplitudes at beat and meter frequencies (all *p*'s > 0.255). Interestingly, reported hours of music listening also did not correlate with years of combined parent music training, *p* = 0.579, and did not differ across infant music class groups, *p* = 0.968

## Experiment 2

### Materials and methods

#### Participants

Thirty-three infants between the ages of 14- and 16-months (17 males; *M* age = 15.45 mo, SD = 0.79 mo) participated in this experiment. An additional 2 infants participated, but were too fussy to complete the experiment and were not included in the analyses. The results reported here for Experiment 2 are subsets of results from a larger study on the effect of infant music training. Here, we only report on the EEG portion of the experiment. These infants were recruited when they were between 9- and 10-months-old, and were randomly assigned to either a music training condition or a control condition. Infants in the music training condition (*n* = 14) received 20 weeks (1 h a week) of caregiver-infant music classes, provided by the Royal Conservatory of Music in Hamilton, ON. Infants in the control condition (*n* = 19) received this training after all experimental testing procedures were complete, so they had not received music training at the time of testing. EEG data collection took place within 2 weeks following the 20-week music training period. These infants were recruited from the Developmental Studies Database at McMaster University. The McMaster Research Ethics Board approved all procedures and informed consent was obtained from parents.

#### Stimulus

**Figure 4:**
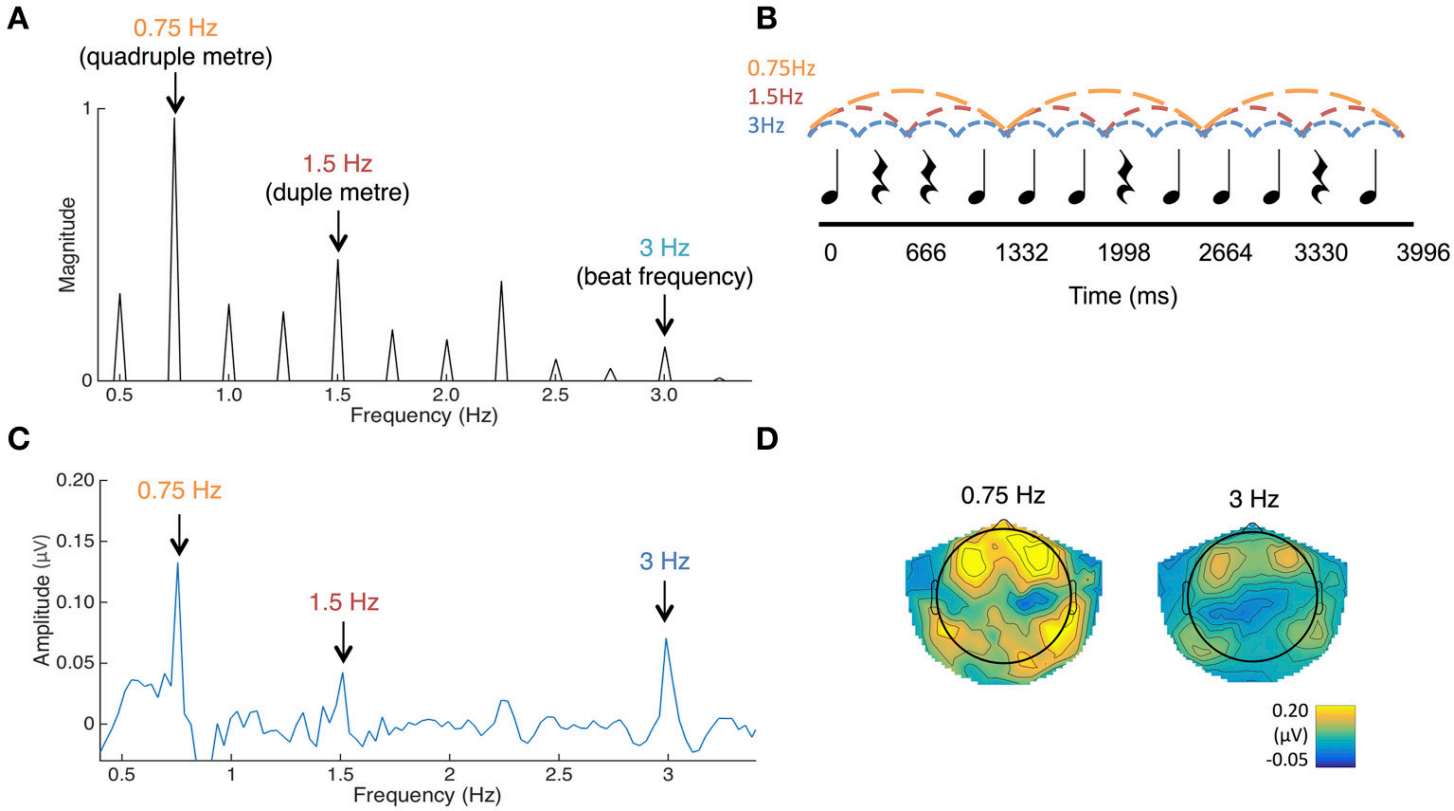
**(A)** The frequency spectrum of the stimulus sound envelope. **(B)** The rhythmic pattern, which consisted of 333 ms long tones and rests. Each tone consisted of 990 Hz pure tones with 10 ms rise and fall times. **(C)** Average SS-EPs with noise subtracted amplitudes averaged across all channels. Peaks can be visualized at the quadruple meter frequency (0.75 Hz) and beat frequency (3 Hz). **(D)** Average topographical map of the SS-EPs elicited at the quadruple meter frequency (0.75 Hz) and beat frequency (3 Hz), averaged across all participants.

The stimulus consisted of a rhythmic pattern lasting 3.996 s, made up of a rhythmic combination of 12 sounds and silent intervals (Figure 4B). This stimulus was based on the one used by Nozaradan et al. (2012b, 2016). Each beat had an inter-onset-interval of 333 ms (180 beats per minute), which translated to a beat frequency of 3 Hz. The tones were 990 Hz pure tones lasting 333 ms with a 10 ms rise and fall time synthesized using the program Audacity 2.0.5 (www.audacity.sourceforge.net). One 36-s long trial consisted of 9 repetitions of this stimulus. These trials were repeated 14 times, with no pauses between trials, so that the entire procedure lasted about 9 min with no break. The stimulus was presented at a comfortable intensity level [~60 dB SPL over a noise floor of < 30 dB(A)] using Eprime software through an AudioVideo Methods speaker (P73) located ~1 m in front of the infant.

The envelope spectrum of this sequence was analyzed using the same procedure as in Experiment 1, in order to compare stimulus and SS-EPs frequency spectra.

#### Procedure

The procedure matched Experiment 1, except that a table was placed in front of the infant so that they understood that they could not get down and play on the floor. It takes some effort to get infants of this age to sit still, so they were given toys to play with on the table if necessary.

#### Data acquisition and analyses

Data acquisition and analysis matched Experiment 1 in all respects except in epoch length, due to the different stimulus lengths (14 trials of 35,000 ms). The ERPs to the first tone in each of the 12-beat sequences were also analyzed using the same procedure as in Experiment 1 (total trials = 146).

### Results

The z-score cutoff method described in Experiment 1 was employed here, using the average SS-EP amplitude across all frequencies tagged from the stimulus. No infants met the ±3 z-score cutoff criteria, and so all were included in the following analyses.

#### SS-EP responses

SS-EPs averaged across all channels and scalp topographies are visualized in Figures 4C,D. The expected beat frequency is at 3 Hz (as tones and rests in the rhythm pattern are 333 ms long). Based on previous work with this rhythm pattern (Nozaradan et al., 2012b, 2016), these beats are most naturally grouped in 4's, representing the quadruple meter at 0.75 Hz. Grouping these beats in 2's (1.5 Hz) is also fairly common. To determine if the peaks in the frequency-transformed EEG occur as expected above the noise floor, peaks of interest were first determined from the FFT of the sound stimulus (See Figure 4A). Amplitudes in the frequency-transformed EEG were calculated at frequencies where peaks were present in the sound stimulus (0.75, 1, 1.25, 1.5, 1.75, 2, 2.25, 2.5, 2.75, and 3 Hz; 0.5 Hz was excluded due to our use of a 0.5 Hz high pass filter) and at frequencies between these, where no peaks were present in the sound stimulus (0.625, 0.875, 1.125, 1.375, 1.625, 1.875, 2.125, 2.375, 2.625, and 2.875 Hz). These amplitudes were calculated by selecting the maximum amplitude within a 3-bin band centered on each frequency of interest.

Noise floor amplitude was calculated as the average across the frequencies not present in the sound. Using paired-samples *t*-tests corrected for multiple comparisons with the Bonferroni correction, EEG amplitudes at each of the frequencies contained in the sound stimulus were compared to this noise floor value. Amplitudes at 0.75 Hz [*t*_(32)_ = 6.54, *p* < 0.001] and 3 Hz [*t*_(32)_ = 4.49, *p* < 0.001] were significantly greater than the noise floor value. Amplitudes at 1.5 Hz were also significantly greater than the noise floor [*t*_(32)_ = 2.80, *p* = 0.009], but this was not significant using the Bonferroni corrected alpha value of *p* < 0.005 for each comparison. No other amplitudes at frequencies present in the stimulus were significantly larger than the noise floor value. In addition, the average amplitude of beat and meter-related frequencies (0.75, 1.5, 3 Hz) was significantly greater than the average amplitude of beat- and meter-unrelated frequencies present in the sound (1, 1.25, 1.75, 2, 2.25, 2.75 Hz), *t*_(32)_ = 7.806, *p* < 0.001. Together, these results suggest that the frequency tagging SS-EP method can result in good signal to noise ratios when used with this age group.

#### Effects of music background

##### Effects of infant music classes

An ANOVA with participation in music classes as a between subjects variable and frequency (only those that were above the noise floor, i.e., 0.75, 1.5, and 3 Hz) as a within subjects variable was used to investigate SS-EP amplitudes. Surprisingly, there was no main effect of infant music classes [*F*_(1, 31)_ = 0.09, *p* = 0.761] and no interaction between music classes and frequency [*F*_(1.48, 45.76)_ = 0.17, *p* = 0.778]. This suggests that the 20 weeks of music training provided to the experimental group may not have influenced this measure

##### Effects of parent music training

Years of parent music training were calculated as a combination of mother and father reported levels, as in Experiment 1. Infants were divided into two groups based on this information: infants with parents who had ≥5 years of music training (*n* = 16), and infants with parents who had < 5 years of music training (*n* = 17)

**Figure 5:**
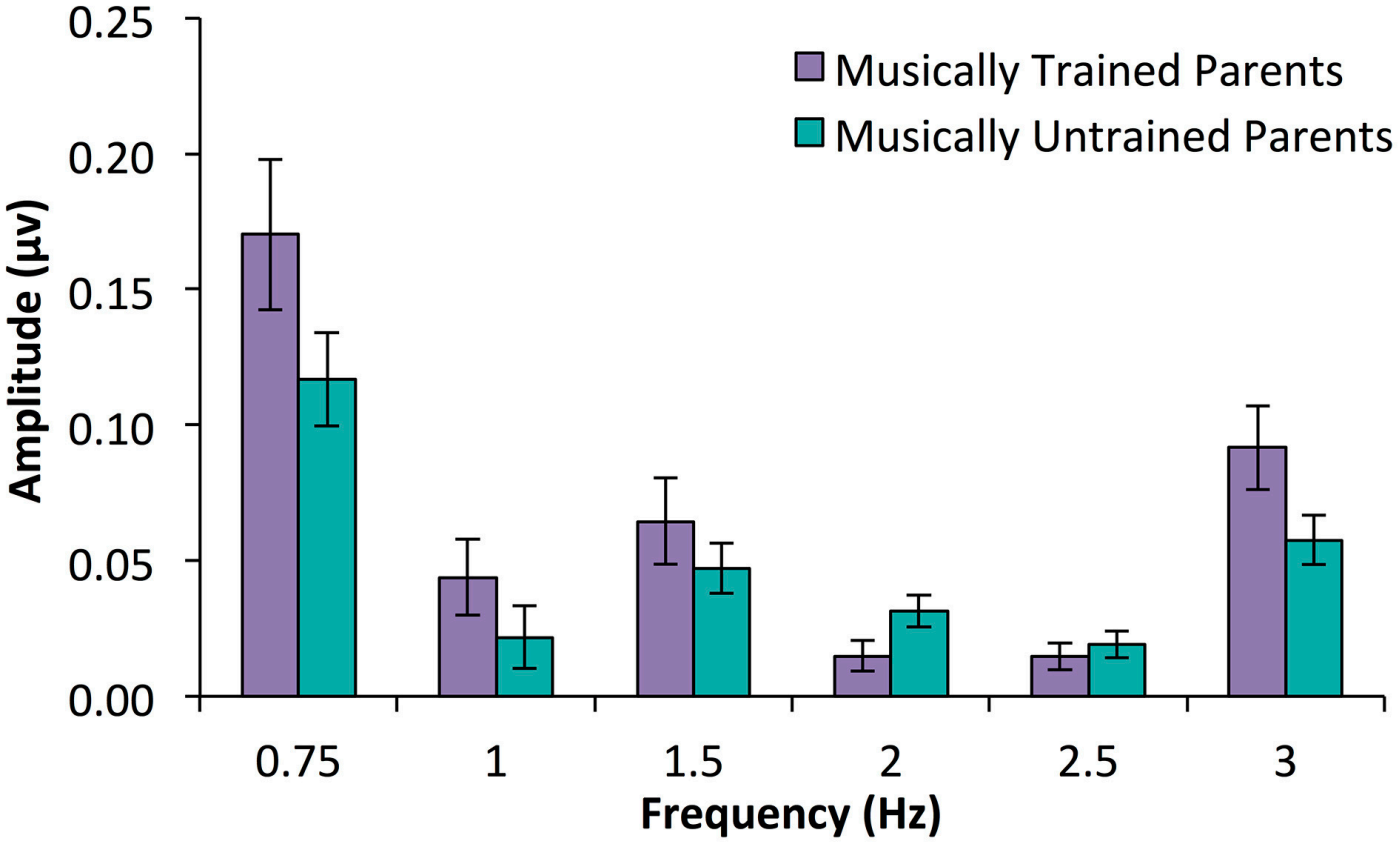
**SS-EP amplitudes (noise subtracted, averaged across all channels) across frequencies of interest for 15-month-old infants who have parents with ≥5 years of combined music training (***n*** = 16, musically trained parents) and infants with < 5 years of combined parent music training (***n*** = 17, musically untrained parents)**. Infants with musically trained parents have larger peaks across SS-EP frequencies compared to infants without musically trained parents, *p* = 0.037.

In an ANOVA with parent music training group as a between subjects variable and frequency (only those that were above the noise floor, i.e., 0.75, 1.5, and 3 Hz) as a within subjects variable, a significant main effects of parent music group was found, *F*_(1, 31)_ = 4.73, *p* = 0.037. There was also a main effect of frequency [*F*_(1.46, 45.27)_ = 19.52, *p* < 0.001] driven by the fact that responses at 0.75 Hz were larger than responses at 1.5 or 3 Hz. Interestingly, there was no interaction between parent music training and frequency, *F*_(1.46, 45.27)_ = 0.76, *p* = 0.473. These results suggest that infants with musically trained parents had larger average SS-EP amplitude overall compared to infants with musically untrained parents (See Figure 5).

**Figure 6:**
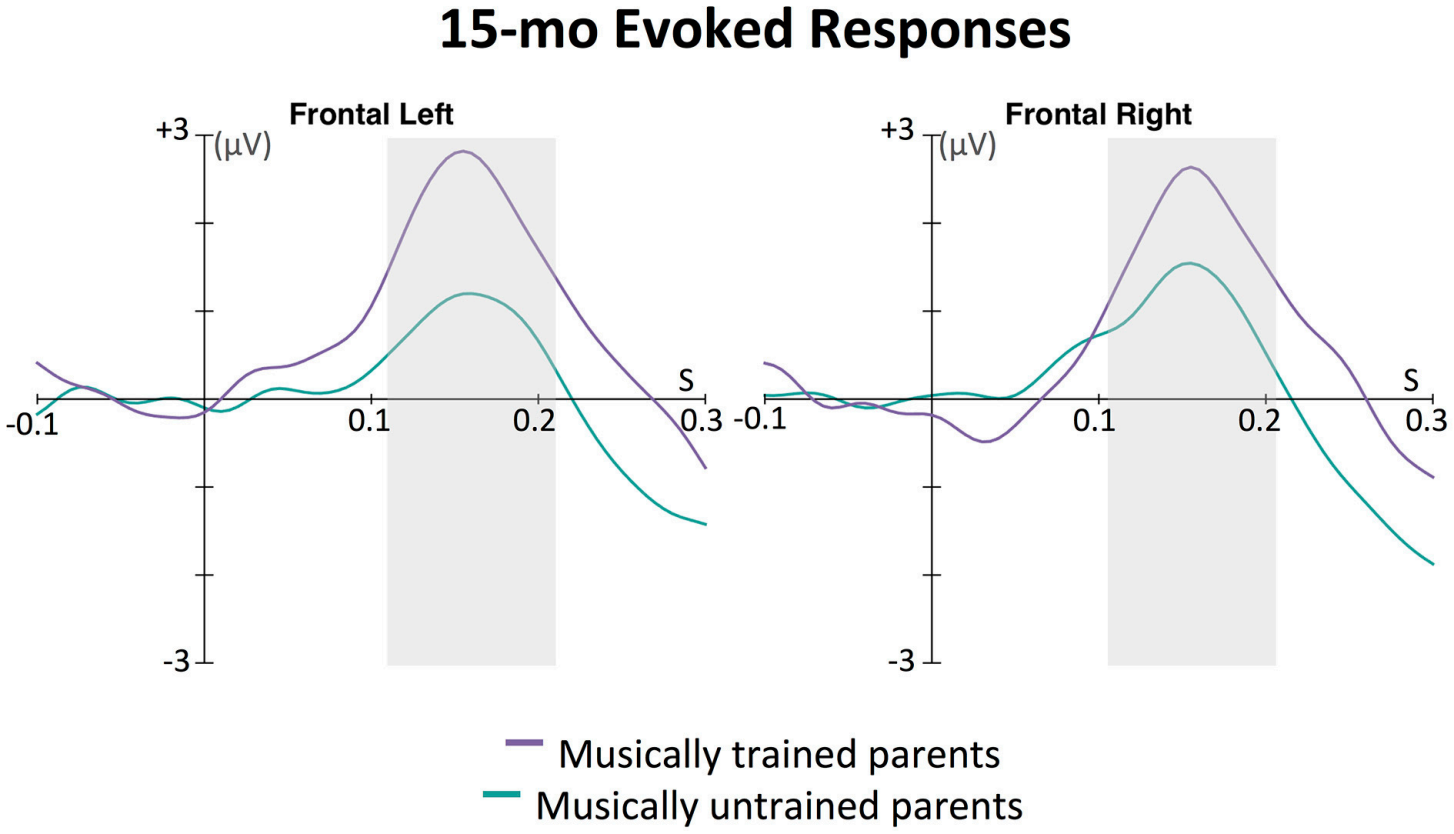
**Grand average evoked responses (filters: 0.5 Hz highpass, 20 Hz lowpass) by 15-month-old infants to the first tone following a silence in the 12-beat sequence, shown in the left and right frontal grouping locations**. The magnitude of the response for each group for each hemisphere was calculated as the area under the curve in a window ±50 ms around 160 ms for the left and 155 ms for the right hemisphere (shown with the gray boxes); these were the time points of the largest amplitude peak in grand average of the slow positive evoked responses. In an ANOVA with parent music training (high, low) as a between-subjects factor and hemisphere (left, right) as a within-subjects factor, only a main effect of parent music training was found. Infants with musically trained parents showed larger positive evoked responses to the sounds than infants without musically trained parents, *p* = 0.025.

This effect was further explored in the ERPs. As can be seen in Figure 6, this wave peaked around 160 ms after tone onset for the left frontal, and 155 ms after tone onset for the right frontal location. The area under the curve method described above was used to assess the magnitude of this auditory response. An ANOVA was conducted with between-subjects factor parent music training (high, low) and within-subjects factor hemisphere (left, right). While there was no main effect of hemisphere [*F*_(1, 31)_ = 0.01, *p* = 0.938] or interaction between hemisphere and parent music training [*F*_(1, 31)_ = 1.24, *p* = 0.275], there was a significant main effect of parent music training, *F*_(1, 31)_ = 5.56, *p* = 0.025. Infants whose parents had more music training had larger evoked responses to these tones compared to infants whose parents had less music training. Supporting this, a positive correlation was found between frontal ERP response magnitude (averaged across hemisphere) and parent years of music training, *r* = 0.47, *p* = 0.006.

##### Reported hours of infant music listening

There were no correlations with reported hours of infant music listening and amplitudes at any of the frequencies of interest (all *p*'s > 0.314). Again, there was surprisingly no correlation between reported hours of music listening and years of parent music training, *p* = 0.236.

## Discussion

Here we reported the results from two infant experiments where we measured SS-EP responses that were frequency-locked to auditory rhythm patterns. In Experiment 1, 7-month-old infants listened to a rhythm pattern that could either be interpreted as triple or duple meter. Infants showed obvious amplitude peaks at the beat level (3 Hz) as well as both potential meter levels (duple and triple meter frequencies). Interestingly, infants who had music training showed greater SS-EP amplitudes elicited at duple meter (but not beat) frequencies than infants who had not received music training. ERP analyses also revealed that these infants with music training had larger evoked responses to the first tone in the rhythmic pattern than the infants with no training. In Experiment 2, 15-month-old infants listened to a rhythm pattern that could easily be interpreted as quadruple meter. Again, in an even shorter testing period (9 min), we found clean signal-to-noise ratios with clear amplitude peaks at both the beat (3 Hz) and meter (0.75 and 1.5 Hz) frequencies. While we did not find an influence of infant music training on SS-EP amplitudes with this age group, we did find an interesting effect of parent music training. Infants with parents who had at least 5 years of combined music training showed larger amplitudes across SS-EP frequencies than infants without musically trained parents. ERP analyses also revealed that babies with musically trained parents had larger evoked responses to the first tone in the sequence than the infants with musically untrained parents. We also found a positive correlation between parent years of training and ERP magnitude.

These results highlight the usefulness of the frequency tagging method for testing rhythm processing in infants with EEG. In both age groups (7 and 15 months), with relatively short testing periods (18 and 9 min, respectively), clear responses above the noise floor were seen at frequencies corresponding to the rhythm envelope. This result provides direct evidence for the capacity of the infant's brain to entrain, that is, frequency-lock, to incoming auditory rhythms. How much of these responses are due to low-level processes of the acoustic inputs and how much is due to top-down perceptual processes remains to be clarified. In adults, active attentional processes and body movement have been shown to shape such responses (Chemin et al., 2014; Nozaradan et al., 2011, 2015). As well, individual differences in adults' tapping ability are reflected in the size of SS-EPs elicited at beat frequencies (Nozaradan et al., 2016). In the present study, while we could not explicitly control the active or automatic attention of the infants, we did find evidence for individual differences in rhythm processing. In Experiment 1 in particular, the effect of infant music training on duple meter frequency peaks, but not beat, frequency peaks, implies that music training may not only selectively amplify specific frequencies, but may also enhance metrical processing. This supports the idea that the SS-EPs we measured are not simply stimulus driven, and may be influenced by higher-level processing. Overall, these experiments suggest that the frequency-tagging method is apt to investigate the mechanisms through which the neural encoding of rhythms is shaped during early development.

Importantly, the frequency-tagging approach appears promising for observing the developmental trajectory of neural entrainment across infancy. The robust signal-to-noise ratios obtained in 7- and 15-month-olds bode well for observing effects as young as the newborn period. Little is known about rhythm processing in very young infants, and the frequency tagging approach offers a potential way to study this. Furthermore, it could be used in conjunction with future experimental designs in which infant musical experience is controlled through random assignment to different types of training. The effects of infant music training in Experiment 1 (where assignment to training was not controlled) and lack of effects of infant music training in Experiment 2 (where all parents enrolled their infants in music classes, but the classes were delayed in the control group so that those infants were untrained at the time of testing) suggest that either (1) training must occur early, before musical enculturation, for clear rhythm processing differences to be measured or (2) parents who choose to enroll their children in infant music classes may be different in some related variable from parents who do not.

The individual differences observed in the current study provide new insights on how infant and parent music background might shape music listening experiences. With two age groups and two different rhythm patterns, we found two different relations between music background and neural responses to rhythms. With the younger but not older infants, infant music training was related to enhanced duple meter processing. With older but not younger infants, parent music training was related to a non-specific enhancement across beat and meter frequencies. It is difficult to compare the results across these two experiments given the methodological differences. Here we present possible ideas for why we may have found differences in experiential effects between these age groups, but all such interpretations must be treated with caution. It could be that direct musical exposure in the form of infant music classes is more likely to shape meter processing in younger infants, since they have not yet become fully encultured to the metrical structures of the music in their environment, which occurs between 6 and 12 months (Hannon and Trehub, 2005a,b). It could also be the case that parents with higher levels of music training, through an interplay of genes and environment, encourage their infants to attend to temporal information in music more than parents with less music training. Further research is needed to directly assess these possibilities. Other differences in parent-infant lifestyles across these groups (SES, parent involvement in infant's daily life) would also need to be measured and controlled.

It was surprising that a correlation between reported parent years of training and infant music listening was not found in Experiments 1 or 2. Previous work has shown that parents with more music training typically engage in more musical activities with their babies. More specifically, parental musical experience was associated with the habit of listening to music with baby (Ilari, 2005), and with the frequency of playing music to and singing to baby (Custodero and Johnson-Green, 2003). It is possible that our simple question “How many hours per week does your infant hear music” (which covers both active and passive listening, with and without the parent) was not specific enough to capture potential differences in levels of musical engagement between parent and infant. Therefore, it is possible that our measure of parent music training is a better proxy for infant exposure to music in engaged settings than the question in our questionnaire.

One limitation of this study is that parents did not wear noise-canceling headphones. It is possible that some mothers (despite being blind as to our hypotheses and despite our clear instruction to avoid movement) may have subtly moved their bodies (and therefore their baby) to the rhythms heard over the loudspeaker, which may have influenced EEG recordings. The experimenter in the room tasked with infant distraction was trained to watch for such movements and, should they occur, to communicate to the parent that they must stop. All parents complied with this instruction. We also had a second experimenter watching the parent and infant from outside the sound attenuated chamber via a live webcam feed, to ensure that instructions were being followed.

Overall, the results of these experiments provide new insights on how the processing of beat and meter may develop from the interplay between genes and environment. Specifically, we present evidence that the frequency-tagging approach is apt to measure infants' neural entrainment to rhythmic patterns. We also present evidence that the neural responses entrained to beat and meter frequencies can be influenced by individual differences in infant and parent music backgrounds. These findings raise interesting questions about how musical experiences across the lifespan, especially in the early months of infancy, shape auditory processing in general and temporal processing in particular.

## Author contributions

LC was the primary researcher and LT the senior researcher but all authors contributed to the ideas, analyses, and writing of the manuscript. LC and CS tested participants.

### Conflict of interest statement

The authors declare that the research was conducted in the absence of any commercial or financial relationships that could be construed as a potential conflict of interest.
